# The impact of maternal high-fat diet on offspring neurodevelopment

**DOI:** 10.3389/fnins.2022.909762

**Published:** 2022-07-22

**Authors:** Gintare Urbonaite, Agne Knyzeliene, Fanny Sophia Bunn, Adomas Smalskys, Urte Neniskyte

**Affiliations:** ^1^Institute of Biosciences, Life Sciences Center, Vilnius University, Vilnius, Lithuania; ^2^Centre for Cardiovascular Science, The Queen’s Medical Research Centre, The University of Edinburgh, Edinburgh, United Kingdom; ^3^Faculty of Science and Engineering, University of Groningen, Groningen, Netherlands; ^4^VU LSC-EMBL Partnership for Genome Editing Technologies, Life Sciences Center, Vilnius University, Vilnius, Lithuania

**Keywords:** maternal high-fat diet (mHFD), inflammation, gut microbiota, epigenetic regulation, behavioral deficits, neurodevelopmental disorders

## Abstract

A maternal high-fat diet affects offspring neurodevelopment with long-term consequences on their brain health and behavior. During the past three decades, obesity has rapidly increased in the whole human population worldwide, including women of reproductive age. It is known that maternal obesity caused by a high-fat diet may lead to neurodevelopmental disorders in their offspring, such as autism spectrum disorder, attention deficit hyperactivity disorder, anxiety, depression, and schizophrenia. A maternal high-fat diet can affect offspring neurodevelopment due to inflammatory activation of the maternal gut, adipose tissue, and placenta, mirrored by increased levels of pro-inflammatory cytokines in both maternal and fetal circulation. Furthermore, a maternal high fat diet causes gut microbial dysbiosis further contributing to increased inflammatory milieu during pregnancy and lactation, thus disturbing both prenatal and postnatal neurodevelopment of the offspring. In addition, global molecular and cellular changes in the offspring’s brain may occur due to epigenetic modifications including the downregulation of brain-derived neurotrophic factor (BDNF) expression and the activation of the endocannabinoid system. These neurodevelopmental aberrations are reflected in behavioral deficits observed in animals, corresponding to behavioral phenotypes of certain neurodevelopmental disorders in humans. Here we reviewed recent findings from rodent models and from human studies to reveal potential mechanisms by which a maternal high-fat diet interferes with the neurodevelopment of the offspring.

## Introduction

Obesity is a growing health issue for the human population worldwide (World Health Organization, 2021). Due to the consumption of high-fat diet (HFD) and Western-type diet (Ahima, 2011), the number of overweight and obese adults has exceeded more than 1.9 billion in 2017–2018, representing 42.5% of the United States population (Fryar et al., 2020), 64% of Canada population (Government Of Canada, 2018), and 53% of the European population (EUROSTAT, 2021). If the global trend continues, NCD Risk Factor Collaboration predicts that the prevalence of obesity in 2025 could reach 21% for women and 18% for men worldwide, leading to both serious health issues as well as social and economic consequences (NCD Risk Factor Collaboration, 2016).

Notably, the rates of overweight and obesity are also increasing among women of reproductive age (Kim et al., 2007), leading to metabolic disorders, type-2 diabetes, and gestational diabetes – conditions that during pregnancy may affect the development of the embryo and the fetus. For example, in 2014, in England and Wales, diabetes has been diagnosed in 5% of pregnant women, of which 87.5% had gestational diabetes (Health and Excellence, 2015). Importantly, maternal obesity and metabolic disturbance during pregnancy are critically associated with the metabolic programming of the offspring, both in humans and in animal models (Pinney and Simmons, 2012; Poston, 2012). In addition, maternal metabolic disorders caused by HFD alter offspring brain development leading to neurodevelopmental disorders, such as autism spectrum disorder (ASD) (Krakowiak et al., 2012; Moss and Chugani, 2014; Reynolds et al., 2014b; Li et al., 2016), attention deficit hyperactivity disorder (ADHD) (Rodriguez et al., 2008; Rodriguez, 2010; Buss et al., 2012; Chen et al., 2014b), anxiety and depression (Rodriguez, 2010; Van Lieshout et al., 2013), or schizophrenia (Schaefer et al., 2000; Kawai et al., 2004; Khandaker et al., 2012; Rivera et al., 2015). Acknowledging the burden of such disorders, a wide community of researchers aims to delineate their potential mechanisms and identify possible therapeutic strategies to alleviate them. These studies often rely on animal models that provide a platform for molecular studies within restricted timelines (Kleinert et al., 2018).

**FIGURE 1:**
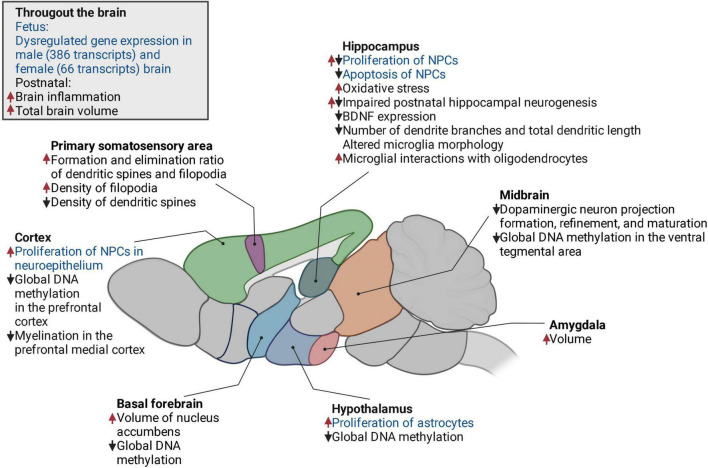
The changes in the offspring brain induced by mHFD in a mouse model. Increase, upward red arrow; decrease, downward black arrow; fetal, text in blue; postnatal, text in black. NPCs, neural progenitor cells; BDNF, brain-derived neurotrophic factor.

Therefore, in this review, we first describe rodent models for maternal high-fat diet (mHFD). Current data suggest that mHFD affects the development of the brain leading to both prenatal deficits and long-term effects that are sustained until adulthood. Such changes are observed globally throughout the brain as well as locally in specific brain regions (Figure 1). Throughout this review, the evidence from rodent models is compared with the human data aiming to suggest potential pathways through which mHFD can impair offspring neurodevelopment in humans.

## Maternal high-fat diet models

To date, many animal models have been developed to explore the effects of mHFD on offspring neurodevelopment (Kleinert et al., 2018). Small rodents, including rats and mice, are the most widely used to study mHFD-related disorders due to their physiological relevance to human conditions. There is a wide range of molecular tools available for rodents and due to the short life span, the effects of mHFD can be evaluated for several generations (Fusco et al., 2019). Moreover, behavioral phenotyping of rodents is well standardized and there is abundant equipment to assess different deficits that can be compared to behavior patterns in humans. Preclinical animal models help to identify the mechanisms and causes of pathology. However, in order to improve the translation from rodents to humans, researchers must not only carefully select the appropriate rodent species and strains, but also regarding the composition of HFD and the timeline of its consumption. These aspects are very important when planning robust experiments and comparing the results with published data of other studies. Therefore, this section will review the mouse models used to study mHFD effects on offspring neurodevelopment.

### The fat in the diet

For humans, it is recommended to get approximately 20–35% of energy from fat (Manore, 2005). However, currently, the typical modern human diet contains about 35–40% fat by energy and can reach up to 60% fat by energy (Speakman, 2019). Human obesity is modeled in rodents by feeding them HFD consisting of 40–60% of the energy derived from fat. Commercial suppliers usually suggest two types of diets, with a fat content of either 45 or 60%, both of which can cause obesity in rodents. In correspondence to the absolute fat content in the diet, it would appear that 45% HFD for rodents is more relative to human HFD and may resemble human physiology better (Speakman, 2019). Nonetheless, due to faster and higher weight gains, which reduces the duration and the cost of the studies, rodent 60% HFD is also often used (Speakman, 2019).

It is important to keep in mind that for rodents 45 and 60% fat diets may not be equivalent and may cause different metabolic responses. For example, in mice, a 60% fat diet has been shown to cause larger weight gain, disrupt glucose homeostasis, and increase insulin resistance to a greater extent compared to a 45% fat diet (Takahashi et al., 1999; Hu et al., 2018). Yet other studies have failed to identify such differences (Mundy et al., 2007; Morrison et al., 2010; Arnold et al., 2014). Alterations in the metabolome induced by an obesogenic diet may also depend on the content of fat. For example, it has been reported that out of 80 metabolites significantly altered in mouse lungs due to HFD, less than half of them (35) were shared by 45 and 60% fat groups, while others were specific to either 45 or 60% HFDs (13 and 32 metabolites, respectively) (Showalter et al., 2018).

In addition to the amount of fat, metabolic response to HFD also depends on the origin and type of fat, which may vary in different fatty diets (Buettner et al., 2007; Stott and Marino, 2020). Therefore, it is important to take into account the ratio of saturated, monounsaturated, and polyunsaturated fatty acids, particularly when investigating the effects of mHFD on offspring health and neurodevelopment. Most the rodent HFDs contain a higher amount of saturated fat (lard, butter, milk, hydrogenated coconut oil, corn oil, palmitic acid, and stearic acid) than unsaturated fat (soybean, olive, and fish oil) (Buettner et al., 2007; Stott and Marino, 2020). A maternal diet high in saturated fat may result in decreased hippocampal size in mice offspring (Niculescu and Lupu, 2009). These HFDs are usually low in polyunsaturated fatty acids (PUFA), such as omega-3 fatty acids, which are essential during prenatal development. PUFA deficiency has been shown to alter the morphology and function of hippocampal neurons and increase microglial phagocytosis of synaptic elements in the developing hippocampus, leading to abnormal hippocampal neural network formation in the offspring (Madore et al., 2020). In mice, maternal diets high in omega-6 and low in omega-3 fatty acids, which reflect modern human diet (such as the Western pattern diet), may lead to impaired neocortical neurogenesis, resulting in long-lasting effects on the offspring brain (Sakayori et al., 2016). For instance, maternal consumption of a diet high in omega-6 during pregnancy and lactation has been shown to increase anxiety and decrease sociability in the mice offspring (Jones et al., 2013). In rats, omega-3-deficiency in the maternal diet causes irreversible changes in the fatty acid composition of the hippocampus, decreases the expression of nerve growth factors (Ikemoto et al., 2000), and disturbs dopamine production (Kodas et al., 2002). Thus, the fatty acid composition should also be considered when selecting a diet for the mHFD model, as any discrepancies in the omega-6-to-omega-3 ratio may impact neurodevelopmental response.

### The choice of rodent species and strains for maternal high-fat diet models

Both rats and mice have been used to study mHFD effects on offspring neurodevelopment. Even though such studies are similarly designed, the described phenotypes are still variable. This could be explained by the fact that the effects of HFD depend not only on dietary components, energy by fat, duration of the diet, and technical variability in different laboratories (Lai et al., 2014), but also, on the selected animal species or strains. The choice of rodent species and strains in HFD studies has been discussed in detail in a number of previously published review articles (Buettner et al., 2007; Stott and Marino, 2020; E Dias et al., 2021; Gawlińska et al., 2021). Among the rat models, Sprague Dawley and Wistar outbred rats are the most popular strains in such types of studies (Lauterio et al., 1994). Regarding the mice, only a few studies have used outbred strains (such as Swiss or CD-1) to model mHFD (Kruse et al., 2013; Marei et al., 2020; Moazzam et al., 2021). However, none of these studies evaluated the consequences of mHFD on the neurodevelopment of the offspring. As outbred mouse strains are genetically heterogeneous, they may be more comparable to the intrinsically heterogeneous human population (Kebede and Attie, 2014). Therefore, it would be beneficial to design studies to investigate the neurodevelopment of mHFD offspring in outbred strains as well. Such studies would reveal more general effects of mHFD that are independent of specific genetic variants in inbred mouse strains and are more translatable to the human population. For now, mHFD studies usually involve inbred strains only. The dams and offspring from such strains share genetic uniformity, therefore limiting the variability of obtained results. Importantly, some inbred strains are more susceptible to HFD-induced obesity than others and it has been suggested that the most appropriate strains for diet-induced obesity studies are C57BL/6J [and its substrains (Siersbæk et al., 2020)] and AKR/J (West et al., 1992; Rossmeisl et al., 2003). Therefore, most studies on mHFD effects on offspring neurodevelopment are performed using C57BL/6J mouse strain. In this strain, HFD elicits similar responses as in humans, including obesity, hyperinsulinemia, hyperglycemia, and hypertension, while animals on a control diet remain lean and healthy (Gregoire et al., 2002; Collins et al., 2004; Wang and Liao, 2012; Calligaris et al., 2013; Loria et al., 2017).

### Study design for mice maternal high-fat diet models

To investigate the effect of mHFD on offspring neurodevelopment, dams are usually fed HFD from 3 to 8 weeks of age for 9–20 weeks (Niculescu and Lupu, 2009; Tozuka et al., 2009, 2010; Kang et al., 2014; Vogt et al., 2014; Edlow et al., 2016; Hatanaka et al., 2016; Glendining and Jasoni, 2019; Fernandes et al., 2021). In most cases, the period of HFD includes the time before mating as well as pregnancy and lactation (Niculescu and Lupu, 2009; Tozuka et al., 2009, 2010; Edlow et al., 2016; Hatanaka et al., 2016; Glendining and Jasoni, 2019; Fernandes et al., 2021). The evidence suggests that the most critical period of maternal consumption of HFD may be lactation (Kang et al., 2014; Vogt et al., 2014; Lippert et al., 2020). For example, the change of maternal diet from high fat to normal exclusively during lactation has been shown to reduce inflammation of the brain in female offspring (Kang et al., 2014). Furthermore, maternal consumption of high fat only during lactation can cause impaired axon formation leading to the consistent decrease of ARH neuronal fiber densities in hypothalamic areas in offspring of both sexes (Vogt et al., 2014). In contrast to the dams, mating males for mHFD studies are maintained on a normal diet, in order to exclusively assess the effects of maternal diet (Niculescu and Lupu, 2009; Tozuka et al., 2009, 2010; Kang et al., 2014; Vogt et al., 2014; Edlow et al., 2016; Hatanaka et al., 2016; Glendining and Jasoni, 2019; Fernandes et al., 2021). Even though there are studies that investigate the effects of paternal HFD on offspring neurodevelopment (Korgan et al., 2018; Zhou et al., 2018; Bodden et al., 2020, 2022), this question was beyond the scope of this review.

The neurodevelopment of the offspring is usually evaluated over a wide time interval from the embryonic stage [from embryonic day (E) 17] until adulthood (up to 80 weeks) (Niculescu and Lupu, 2009; Tozuka et al., 2009, 2010; Kang et al., 2014; Buffington et al., 2016; Edlow et al., 2016; Wankhade et al., 2017; Lippert et al., 2020). Functional and morphological evaluation of the brain is usually performed in embryos and juvenile mice (Niculescu and Lupu, 2009; Kang et al., 2014; Edlow et al., 2016). Meanwhile, microbiota and behavioral studies are mostly performed in adults and only occasionally in juveniles and aged animals (Tozuka et al., 2009, 2010; Peleg-Raibstein et al., 2012; Buffington et al., 2016; Graf et al., 2016; Wankhade et al., 2017; Zieba et al., 2019; Zhou et al., 2020). Depending on the aim of the study, the offspring may be weaned either to a normal or a high-fat diet (Kang et al., 2014; Vogt et al., 2014). As weaning to HFD may affect offspring neurodevelopment directly, in this review, we included only those studies, in which the offspring were weaned to a normal diet, in order to focus on the consequences of mHFD.

It is worth noting that many published studies include male offspring only (Niculescu and Lupu, 2009; Tozuka et al., 2009, 2010; Vogt et al., 2014; Buffington et al., 2016; Edlow et al., 2016; Hatanaka et al., 2016). Since there is accumulating evidence on sex differences in neurodevelopment (Giedd et al., 1997; Dearden et al., 2018; Mossa and Manzini, 2021), the results of male studies have limited applicability when aiming to understand the mHFD effect on female offspring (Giedd et al., 1997; Dearden et al., 2018; Mossa and Manzini, 2021). Therefore, it is currently recommended that any studies on preclinical animal models and humans, clinical and cohort human studies include both males and females (White et al., 2021).

## The consequences of maternal high-fat diet to the offspring brain in mice

Maternal high-fat diet affects both prenatal and postnatal neurodevelopment of the offspring, thus molecular, cellular, and morphological changes in the brain have been observed in fetal, juvenile, and adult mice born to dams fed by HFD. Figure 1 highlights the effects of mHFD throughout the brain of the offspring and describes the alterations observed in specific brain regions.

It has been shown that exposure to mHFD increases the total volume of the brain as well as the volume of the medial amygdala and basal forebrain of the offspring (Fernandes et al., 2021). Brain size abnormalities are more expressed in male than female offspring (Fernandes et al., 2021). The volume of the brain may increase due to increased proliferation and decreased apoptosis of neural progenitor cells (NPCs). In mouse male embryos at E17, mHFD increases the proliferation of NPCs in the neurepithelium of the ventricular and subventricular areas of the hippocampus and the cortex (Niculescu and Lupu, 2009). In contrast, NPCs proliferation within the dentate gyrus decreases (Niculescu and Lupu, 2009). Moreover, the apoptosis of NPCs decreases throughout the hippocampus (Niculescu and Lupu, 2009), further contributing to brain expansion. Postnatally, the proliferation and differentiation of hippocampal NPCs are also impaired by mHFD, possibly due to elevated oxidative stress caused by maternal obesity (Tozuka et al., 2010). This oxidative stress may contribute to the decreased expression of brain-derived neurotrophic factor (BDNF), which is required for normal neurogenesis (Tozuka et al., 2010). In addition, BDNF is essential for synaptic plasticity (Park and Poo, 2013), which is also impaired in mHFD models (Tozuka et al., 2010). For example, in the primary somatosensory cortex, mHFD causes the instability of dendritic spines and filopodia and impairs synaptic maturation (Hatanaka et al., 2016).

It was also found that maternal obesity impairs dendritic arborization of postnatally differentiated neurons in young male’s hippocampus, leading to decreased number of branches and shorter dendritic length (Tozuka et al., 2010). In addition to dendritic plasticity, mHFD affects axonal projections. In both males and females, mHFD alters projection formation, refinement, and maturation of melanocortin neurons in the hypothalamus (Vogt et al., 2014) and dopaminergic circuitry in the midbrain (area and substantia nigra) (Buffington et al., 2016; Lippert et al., 2020). Decreased density of dopaminergic projections results in a lower activity of corresponding neurons and reduces the release of dopamine in the dopaminergic midbrain in adult offspring (Lippert et al., 2020). Interestingly, even though dopaminergic circuitry is affected in both male and female offspring, the observed behavioral phenotype has a clear sexually dimorphic profile. These differences have been suggested to arise from gene expression differences in the brain of male and female offspring leading to distinct behavioral phenotypes (Lippert et al., 2020).

In contrast, it appears that at the embryonic stage, brain transcriptome undergoes more changes in male offspring compared to females (Edlow et al., 2016). At E17.5, mHFD has been shown to dysregulate more genes in the brains of males than females (386 vs. 66 transcripts) (Edlow et al., 2016). Such transcriptomic alterations may be caused by epigenetic dysregulation. mHFD-induced epigenetic alterations have also been observed postnatally. Global DNA hypomethylation has been found in the ventral tegmental area (VTA), nucleus accumbens, prefrontal cortex (PFC), and hypothalamus of adult male offspring (Vucetic et al., 2010; Mckee et al., 2018). Interestingly, epigenetic modulation by mHFD appears to have a sexually dimorphic pattern, as mHFD-induced DNA hypomethylation was only observed in males, but not females (Mckee et al., 2018).

In addition to observed neuronal aberrations, mHFD also alters the function of glia cells, such as oligodendrocytes, astrocytes, and microglia (Graf et al., 2016; Kim et al., 2016; Bordeleau et al., 2021). It has been shown that males but not females born to dams fed by HFD had a reduced number of oligodendrocytes and impaired maturation, which may cause hypomyelination in the medial cortex (Graf et al., 2016). Importantly, mHFD may disturb myelination even when oligodendrocyte density, distribution, or maturation remain unaffected, due to oligodendrocyte-microglia crosstalk (Bordeleau et al., 2021). As resident immune cells of the brain, microglia respond to pro-inflammatory cytokines and bacterial metabolites in the circulation and contribute to the inflammatory milieu, thus mediating neurodevelopmental effects of mHFD-induced inflammation and dysbiosis (Huuskonen et al., 2004; Macfabe, 2012; Murabayashi et al., 2013; Reynolds et al., 2014a; Graf et al., 2016; Cowan and Petri, 2018; Lacabanne, 2018; Dias et al., 2020; Talukdar et al., 2020; Davoli-Ferreira et al., 2021). Pro-inflammatory cytokines elevated by mHFD also increase the proliferation of astrocytes, hence indirectly contributing to altered synaptic function and formation (Kim et al., 2016). Overall, the action of pro-inflammatory cytokines is proposed as one of the key mechanisms of mHFD-induced neurodevelopmental disorders.

## Cytokine hypothesis of neurodevelopmental disorders caused by maternal high-fat diet

**FIGURE 2:**
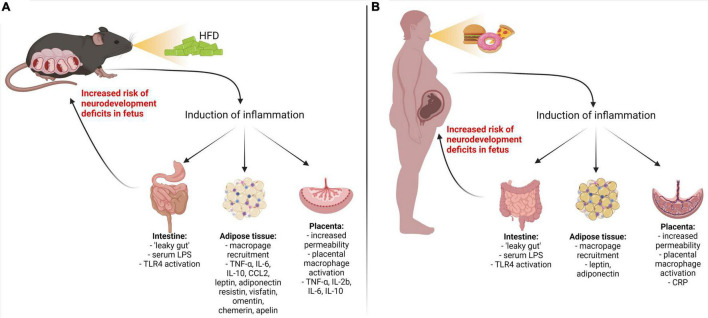
Inflammatory pathways activated by maternal high fat diet-induced metabolic diseases. Metabolic disorder in pregnant mouse dams **(A)** and women **(B)** results in upregulation of pro-inflammatory cytokines/adipokines through the gut, adipose, and placental pathways. LPS, lipopolysaccharide; TLR4, Toll-like receptor 4; IL-1β, IL-6, IL-8, IL-17a – interleukins 1 beta, 6, 8, 17a; TNF-α, tumor necrosis factor-alpha; CRP, C-reactive protein; CCL2, chemokine (C–C motif) ligand; IFN-γ, interferon-gamma.

When unbalanced consumption of HFD leads to the development of metabolic disorders and gut dysbiosis, they are often accompanied by chronic low-grade inflammation (Bruder-Nascimento et al., 2017; Duan et al., 2018; He et al., 2018; Heo and Choung, 2018; Yuan et al., 2018; Lasker et al., 2019) (Figure 2). During inflammation, the immune system produces a range of proteins, collectively referred to as cytokines, that help to fight infections and repair injuries. However, prolonged uncontrolled exposure to pro-inflammatory cytokines is well known to contribute to various pathologies, including metabolic and neurological diseases (D O’brien et al., 2017). Significantly higher levels of cytokines, such as interleukins 1 beta, 2, 5, 6, 10, 12, 13, 17 (IL-1β, IL-2, IL-5, IL-6, IL-10, IL-12, IL-13, and IL-17), interferon-gamma (IFN-γ), and tumor necrosis factor-alpha (TNF-α) have been found in the serum in response to HFD (Kim et al., 2011; Duan et al., 2018). This chronic low-grade inflammation may negatively affect the neurodevelopment of the offspring of mothers consuming HFD (Kim et al., 2014; Sanders et al., 2014; Graf et al., 2016). The evidence from human studies shows a close relationship between prenatal exposure to pro-inflammatory cytokines, such as TNF-α, IL-1β, IL-6, IL-17a, and ASD, schizophrenia, anxiety, depression, and ADHD (Meyer, 2013; Knuesel et al., 2014; Edlow, 2017; Cristiano et al., 2018; Ravaccia and Ghafourian, 2020; Han et al., 2021). Specifically, an increase in maternal IL-6 has been associated with reduced exploratory behavior and deficits in social interaction (Smith et al., 2007). IL-17a may also affect the latter as well as contribute to cortical dysplasia (Choi et al., 2016), whereas IL-1β may cause alterations in white matter development, promote gliogenesis and disturb neurogenesis (Girard et al., 2010; Crampton et al., 2012). These pathophysiological signs are also common among individuals with neurodevelopmental disorders, highlighting the importance of a well-controlled immune environment during gestation. Moreover, animal studies have shown that HFD increases pro-inflammatory cytokines not only in maternal circulation but also in the offspring’s brain, having a direct effect on their neurodevelopment (Bilbo and Tsang, 2010; Kang et al., 2014; Graf et al., 2016; Dias et al., 2020).

Therefore, this section of the review will focus on the findings from animal and human studies and discuss possible links between aberrant cytokine production in HFD females and the neurodevelopmental outcomes of their offspring. It is important to note that a vast majority of the studies discussed herein have reported sex-dependent effects caused by the exposure to prenatal cytokines, with male offspring being more vulnerable than females. Even though this is an extremely important observation that reflects a higher predisposition to neurodevelopmental disorders in the male population (Ramtekkar et al., 2010; Werling and Geschwind, 2013), sexual dimorphism in cytokine action is beyond the scope of this review.

### Evidence of placenta – fetal brain cytokine axis

The placenta is one of the key organs providing metabolic support to the developing fetus. However, as it resides at the maternal-fetal interface, it may also act as a gateway for maternal-derived inflammatory proteins to the fetus. The placenta may increase intrauterine cytokine levels in two different ways: (1) *via* transfer of cytokines from maternal circulation, and/or (2) by the production of cytokines by placental cells. It has been demonstrated that IL-2 and IL-6, but not IL-1α or TNF-α, are able to translocate from maternal to fetal circulation (Zaretsky et al., 2004; Dahlgren et al., 2006; Ponzio et al., 2007), although the evidence is sparse. In addition, linoleic acid, found in HFD, may increase placental permeability by downregulation of cell adhesion proteins in the labyrinth layer and, in turn, contribute to increased maternal-fetal transfer, as demonstrated in mice (Kretschmer et al., 2020). On another hand, the placenta itself has the capacity to generate cytokines by placental macrophages, stromal cells, and trophoblasts (Montes et al., 1995; Jokhi et al., 1997; Steinborn et al., 1998). It has been shown that HFD increases the levels of TNF-α, IL-2b, IL-6, and IL-10 in the placenta and, consequently, in fetal circulation (Kim et al., 2014) with an expected negative impact on offspring neurodevelopment (Challier et al., 2008; Kim et al., 2014). It is important to note, that placenta is a fetal organ and therefore mHFD effects on the placenta-fetal brain axis may depend on the sex of the offspring (Kim et al., 2014; Karakas, 2015; Edlow et al., 2019; Ceasrine et al., 2021). It has been shown that at gestational day 15.5–17.5 the placentas of mHFD male fetuses have higher levels of proinflammatory cytokines and macrophage activation than those of female fetuses, in which mHFD may result in reduced inflammation (Kim et al., 2014). Such dimorphism may be explained by an enhanced male placental immune response that has been observed *in vitro* (Edlow et al., 2019).

It is suggested that HFD-induced placental inflammation may disturb fetal neurodevelopment (Ceasrine et al., 2021). Indeed, clinical studies have reported an increased risk of ASD in the children of mothers with placental inflammation (Straughen et al., 2017). Placental C-reactive protein upregulation was also associated with a higher incidence of ADHD among children (Shao et al., 2020). However, the direct relationship between HFD-induced metabolic disorders, placental inflammation, and neurodevelopmental outcomes in humans and animal models is yet to be investigated.

### Evidence of maternal adipose tissue – fetal brain adipokine axis

Another source of inflammatory proteins is adipose tissue, which is expanded in response to HFD. HFD facilitates the recruitment of macrophages into adipose tissue leading to increased levels of pro-inflammatory cytokines (Bai and Sun, 2015; Lu et al., 2019). Furthermore, HDF stimulates adipocytes themselves, dysregulating the secretion of inflammatory markers called adipokines, i.e., cytokines of adipose tissue (Saberi et al., 2009; Olefsky and Glass, 2010; Khodabandehloo et al., 2016). These include both classical cytokines, such as IL-6, TNF-α, IL-10, or chemokine (C-C motif) ligand 2 (CCL2), as well as leptin, adiponectin, resistin, visfatin, omentin, chemerin, and apelin. It has been shown that HFD leads to the overexpression of classical cytokines, leptin, chemerin, and apelin, while the expression of adiponectin, resistin, and visfatin is reduced (Hotamisligil et al., 1993; Kępczyñska et al., 2013; Murabayashi et al., 2013; Diba et al., 2018; Ding et al., 2018; Lu et al., 2018; Huang et al., 2019; Zhao et al., 2020a; Costa et al., 2021). Maternal hyperleptinemia has been suggested to induce leptin withdrawal in the fetal brain, resulting in the downregulation of leptin receptors and leptin signaling leading to cognitive impairment (Ding et al., 2018). Leptin receptors are expressed across multiple brain regions, such as the cortex, amygdala, hippocampus, thalamus, and hypothalamus, and it has been postulated that aberrant leptin signaling during development may negatively impact functions associated with these brain areas, such as memory, attention, or emotional control (Valleau and Sullivan, 2014; Cortes-Alvarez et al., 2020). However, some clinical studies did not find any effect on the cognitive abilities of the children of women with increased levels of leptin in the plasma (Li et al., 2019). In contrast, disturbed offspring neurodevelopment was associated with decreased maternal adiponectin (Li et al., 2019). Interestingly, the upregulation of several pro-inflammatory adipokines, including omentin, leptin, resistin, and visfatin has also been found in the serum of children with ASD and ADHD (Ghaffari et al., 2016; Yürümez et al., 2018). This indicates that inflammation of adipose tissue may contribute to the cognitive abnormalities during both pre-and postnatal neurodevelopment stages and suggests that adipose tissue-derived inflammatory proteins are important mediators between maternal metabolic status and fetal brain development.

### Evidence of maternal gut – fetal brain cytokine axis

The HFD may influence the disruption of intestinal wall integrity by reducing the production of tight junction proteins (Cani et al., 2008). This, in turn, may lead to the development of metabolic endotoxemia. Metabolic endotoxemia is characterized by an HFD-induced increase in serum levels of endotoxin lipopolysaccharide (LPS), which is abundant in the outer wall of Gram-negative bacteria, such as those found in gut microbiota (Kim et al., 2012; Moreira et al., 2012; He et al., 2018; Nascimento et al., 2020). LPS escapes the gastrointestinal tract *via* disrupted intestinal epithelium and, upon entering the bloodstream, it triggers the activation of type 4 Toll-like receptors (TLR4) that are expressed by the immune, non-immune and dendritic cells (Mohammad and Thiemermann, 2021). Activated TLRs initiate proinflammatory signaling pathways in these cells, resulting in the systemic production of pro-inflammatory cytokines (Kim et al., 2012; Mohammad and Thiemermann, 2021).

An increase in maternal serum levels of LPS has been linked to negative neurodevelopmental outcomes in the offspring. Intraperitoneal injection of LPS (50 μg/kg, single i.p. dose) to pregnant C57BL/10JNju mice induced an increase in TNF-α levels in the fetal brain, downregulation of synaptic pruning and plasticity regulating proteins and, subsequently, ASD-like behavior *via* TLR4 receptor (Xiao et al., 2021). Prenatal treatment with LPS (125 μg/kg in mice; 1.5 mg/kg in rats) also triggered overexpression of IL-1β, IL-6, IL-10, and TNF-α in the placenta and fetal brain, which were related to abnormal microglial numbers, altered cytokine gene expression, anxiety-like behavior and disrupted social behavioral patterns in neonates and adult offspring (Lacabanne, 2018; Talukdar et al., 2020). On another hand, in humans with obesity serum LPS levels are much lower than the doses used in experimental animal models (0.1–0.6 ng/mL) (Radilla-Vázquez et al., 2016). However, it is noteworthy that in humans LPS is much more potent than in rodents (∼10^6^-fold difference in LD_50_) (Radulovic et al., 2018), thus low levels of LPS might be capable of inducing detrimental neurodevelopmental outcomes.

Numerous studies support the hypothesis of maternal metabolic endotoxemia as a plausible mechanism contributing to increased cytokine production and subsequent neurodevelopmental disorders in children of mothers with HFD-induced metabolic disorders. However, even though the direct evidence, evaluating the mHFD, serum LPS levels, and cytokine profile and relating these measures with the incidence of neurodevelopmental abnormalities in animal models is emerging (Grissom et al., 2017; Edlow et al., 2019), human data is still lacking. Therefore, future clinical studies should aim at filling this knowledge gap.

It is important to note that some studies did not observe an increase in maternal serum LPS in HFD mice, even though gut epithelium dysfunction and NF-κB activation were present (Wallace et al., 2019), suggesting that other mechanisms linking maternal HFD and systemic subclinical inflammation may also be present.

## Maternal microbial dysbiosis and offspring brain development

**FIGURE 3:**
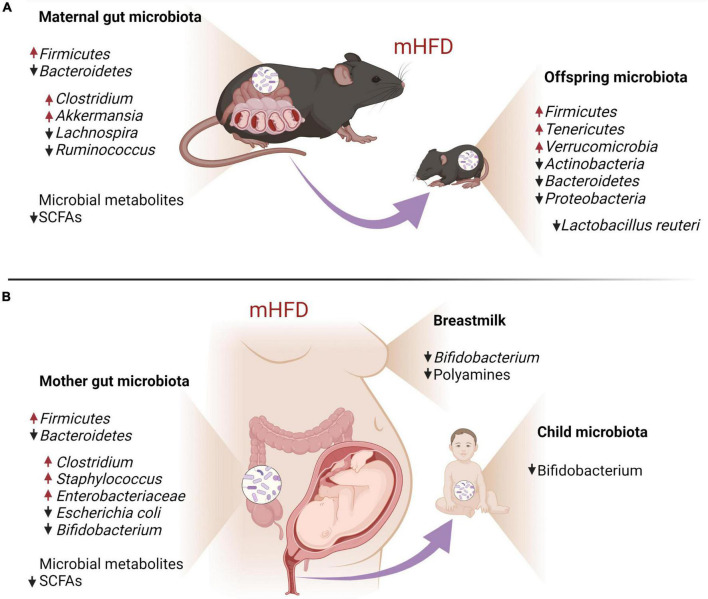
Maternal HFD-induced gut and breastmilk dysbiosis and its impact on offspring gut microbiota in mouse models **(A)** and human individuals **(B)**.

In addition to the abnormal inflammatory milieu, mHFD is also known to change gut microbiota composition in both mother and offspring (Babu et al., 2018). On one hand, altered microbiota can further exacerbate maternal inflammation and impair offspring neurodevelopment due to an imbalance of bacterial metabolites; on the other hand, offspring themselves inherit altered microbiota that further mediate aberrant brain development postnatally (De Theije et al., 2014; Nankova et al., 2014; Babu et al., 2018). Therefore, in this section, we will overview different pathways by which mothers’ gut microbiota interacts with the development of the offspring’s brain prenatally and postnatally (Figure 3).

Consumption of foods that are high in fat changes the gut microbial makeup in both humans and mice. In humans, obesity is associated with an increase in *Firmicutes* and a relatively lower abundance of *Bacteroidetes* (Chen et al., 2014a; John and Mullin, 2016; Tseng and Wu, 2019). Respectively, the gut microbiota of overweight and obese pregnant women also has been shown to have an increased abundance of *Firmicutes* and decreased that of *Bacteroidetes*, together with the increase in *Clostridium, Staphylococcus, Enterobacteriaceae*, and *Escherichia coli* and a decrease in *Bifidobacterium* (Collado et al., 2008; Santacruz et al., 2010; Zacarías et al., 2018). Similarly, pregnant HFD dams exhibit the elevation of *Firmicutes* and the reduction of *Bacteroidetes*, together with a specific increase in *Akkermansia* and *Clostridium* and a decrease in *Lachnospira* and *Ruminococcus* (Takeuchi et al., 1995; Mann et al., 2018; Gohir et al., 2019).

Even though during *in utero* development the fetus has limited interaction with maternal microorganisms due to the placental barrier (Younge et al., 2019), the placenta can be crossed by some bacterial metabolites, such as short-chain fatty acids (SCFAs), the main of which include acetate, propionate, and butyrate (Miller and Wolin, 1996; Louis and Flint, 2009, 2017; Silva et al., 2020). SCFAs have been shown to be essential for the normal development of the fetal brain (Kimura et al., 2020). For example, the decrease of maternal butyrate may contribute to human behavioral and neurologic pathologies, such as depression, later in life (Dalile et al., 2019; Silva et al., 2020). Importantly, in mice, HFD has been shown to reduce the abundance of such genera as *Lachnospira* and *Ruminococcus* (*Clostridia* class, *Firmicutes* phylum), which are required for butyrate production in the gut, leading to reduced butyrate concentration in HFD dams (Gohir et al., 2019). In mammals, SCFAs are recognized by two free fatty acid receptors FFAR3 and FFAR2, also known as GPR41 and GPR43, respectively (He et al., 2020). HFD induces the upregulation of GPR43 (Dewulf et al., 2011) and downregulation of GPR41 (Gohir et al., 2019) in adipose tissue. These receptors are also found in fetal and uteroplacental tissues both in humans and mice (Voltolini et al., 2012; Kimura et al., 2020), suggesting that they can contribute to the maternal gut – fetal brain signaling axis.

Short-chain fatty acids are suggested to modulate neurodevelopment either by directly interacting with neural cells or indirectly through glial cells. *In vitro* and *ex vivo* studies indicate that SCFAs promote the proliferation of human neural progenitor cells (Yang et al., 2020), induce an anti-inflammatory response in primary brain-derived microglial cells and hippocampal slice cultures (Huuskonen et al., 2004), and increase the expression of BDNF and glial cell line-derived neurotrophic factor in astrocytes through histone deacetylase (HDAC) inhibition (Wu et al., 2008). The latter finding suggests that SCFAs are important contributors to brain epigenetic regulation, which will be discussed in detail in the following section.

Maternal microbial dysbiosis may contribute to the postnatal neurodevelopment of the offspring as well. Maternal microbiota is transmitted vertically to the child through the reproductive and gastrointestinal tracts (Favier et al., 2003; Makino et al., 2013), as well as by breastfeeding (Collado et al., 2010). It has been shown that the breast milk of obese mothers has a lower amount of beneficial *Bifidobacteria* that produce acetate, one of SCFAs, which are known to have anti-inflammatory properties (Collado et al., 2010; Fukuda et al., 2011). In addition, breast milk from obese mothers has a significantly lower content of bacterial metabolites polyamines (Ali et al., 2013), which exhibit anti-inflammatory effects as well as modulate the maturation of developing synapses (Pegg, 2016). These changes in breast milk composition may be the reason why lactation appears to be a critical period for the effect of mHFD on offspring neurodevelopment (Kang et al., 2014; Vogt et al., 2014). Maternal diet is one of the main factors determining offspring gut microbiota composition, which further guides neurodevelopment during the postnatal period. mHFD-induced dysbiosis of offspring gut microbiota is associated with reduced bacterial diversity and changes in the relative abundance of certain bacteria species (Galley et al., 2014; Buffington et al., 2016; Wankhade et al., 2017; Guo et al., 2018; Xie et al., 2018). It has been demonstrated, that mHFD increases *Firmicutes*, *Tenericutes*, and *Verrucomicrobia*, and decreases *Actinobacteria, Bacteroidetes*, and *Proteobacteria* in mice offspring (Wankhade et al., 2017; Guo et al., 2018; Xie et al., 2018; Sanguinetti et al., 2019; Zhang et al., 2019). In particular, mHFD has been shown to cause the deficiency of *Lactobacillus* (*L.*) *reuteri* in the offspring gut in mice (Buffington et al., 2016) and *Bifidobacterium* in humans (Guzzardi et al., 2022), mirrored by social and cognitive behavioral deficits. Importantly, mHFD-induced social deficits of the offspring are restored by oral probiotic treatment with lacking commensal *L. reuteri*, indicative of the causal relationship between gut dysbiosis and neurodevelopmental phenotype rather than mere correlation (Buffington et al., 2016). Vagus nerve has been demonstrated to link gut microbiota and the brain of the offspring born to HFD dams (Buffington et al., 2016), and reduced SCFAs levels have been implicated in impaired postnatal neurodevelopment due to microbiota dysbiosis (Liu et al., 2021), but detailed mechanisms are yet to be described.

## Maternal high-fat diet effect via epigenetic regulation

In addition to dysbiosis of gut microbiota inherited by the offspring from HFD mothers, long-term effects of mHFD can also be sustained by altered epigenetic regulation in the offspring’s brain (Vucetic et al., 2010; Glendining and Jasoni, 2019). mHFD modulates prenatal and postnatal offspring neurodevelopment through the changes in histone modifications, DNA methylation, and microRNA expression (Vickers, 2014; Neri and Edlow, 2016). In addition to global DNA hypomethylation, gene-specific epigenetic changes have been described in the brain of mHFD offspring both prenatally and postnatally. For example, higher levels of *oxtr* transcripts in the E17.5 male hippocampus may be caused by increased acetylation in its promoter region (Glendining and Jasoni, 2019). Interestingly, *oxtr* expression has not been affected in females despite decreased promoter methylation, indicating that the outcome of epigenetic changes may be sexually dimorphic (Glendining and Jasoni, 2019). In this section, we will review in detail how mHFD disrupts epigenetic regulation of *Bdnf* expression and the endocannabinoid system in particular, as these systems have critical functions in neurodevelopment.

### Epigenetic regulation of brain-derived neurotrophic factor in the developing brain

Multiple animals and human studies have identified that mHFD contributes to reduced offspring’s hippocampal growth, which can be linked to epigenetic dysregulation of BDNF expression (Eskelinen et al., 2008; Walker et al., 2008; Niculescu and Lupu, 2009; Page et al., 2014). It is well established that BDNF plays a key role in neuronal survival, neurodevelopment, and synaptic plasticity (Ahmed et al., 1995; Bartrup et al., 1997; Stagni et al., 2017). BDNF deficiency due to epigenetic alterations has been linked to a plethora of psychiatric diseases, suggesting that the lack of BDNF is a central biomarker for such disorders (Corominas-Roso et al., 2013; Janke et al., 2015; Jha et al., 2016).

**FIGURE 4:**
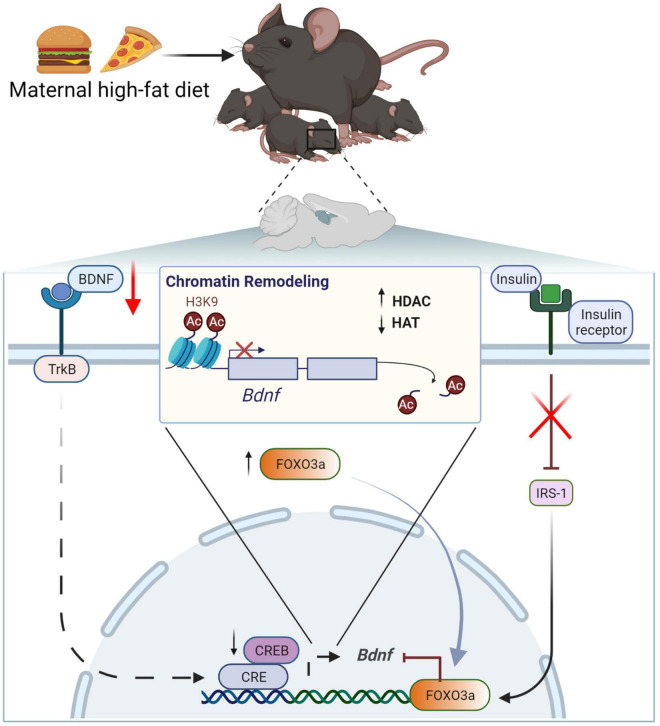
Maternal high-fat diet downregulates BDNF due to chromatin remodeling and increased histone deacetylation on the *Bdnf* promotor. BDNF/*Bdnf*, brain-derived neurotrophic factor; CBP, CREB-binding protein; HDAC, histone deacetylase; HAC, histone acetylase; IRS-1, insulin receptor substrate 1; TrkB, tropomyosin receptor kinase B; CREB, FOXO3a, transcription factors; H3K4, histone H3 lysine 9.

Exposure to mHFD leads to reduced BDNF expression in offspring hippocampus (Tozuka et al., 2010; Fusco et al., 2019). Epigenetic dysregulation of *Bdnf* expression in the offspring’s brain appears to be inherited by imprinting from HFD dams (Fusco et al., 2019). Fusco et al. (2019) have proposed a mechanism, in which HFD-induced dysfunction of maternal metabolism leads to the downregulation of insulin signaling pathway, causing aberrant methylation and acetylation of *Bdnf* promoter in maternal ovaries and transgenerational transfer of epigenetic *Bdnf* downregulation (Figure 4) (Fusco et al., 2019). They demonstrated that insulin resistance is linked to the activation of forkhead box protein O3a (FOXO3a), promoting its translocation to the nucleus and the interaction with chromatin remodelers. In particular, FOXO3a activates HDAC, which deacetylates histones on the *Bdnf* promoter, thus reducing *Bdnf* expression (Fusco et al., 2019). In turn, due to the positive feedback mechanism, low BDNF levels in plasma and ovaries further suppress their own expression. Reduced BDNF signaling through tropomyosin receptor kinase B (TrkB) leads to CREB inhibition and reduced chromatin binding of histone acetylase (HAT), known as CREB-binding protein (CBP) (Fusco et al., 2019). Consequently, histone acetylation on *Bdnf* promoter has been found critically reduced in the ovaries of HFD dams together with reduced histone methylation; however, the mechanism of the latter has not been identified yet (Fusco et al., 2019). Importantly, these epigenetic modifications appear to be inheritable, as they have been identified both in somatic (including hippocampus) and germline cells of the offspring and have been retained for up to three generations (Fusco et al., 2019). It is possible that transgenerational epigenetic imprinting is maintained in the offspring through previously described BDNF auto-regulation *via* TrkB (Fusco et al., 2019).

### Endocannabinoid system changes via an epigenetic mechanism

A maternal high-fat diet can also epigenetically dysregulate the endocannabinoid system in the brain of the offspring (Almeida et al., 2019; Gawliński et al., 2021). Endocannabinoids are recognized by two main receptors, namely cannabinoid receptor 1 (CB1) and cannabinoid receptor 2 (CB2) (Matias and Di Marzo, 2007). CB1 signaling is important for prenatal brain development, regulating neural progenitor differentiation and guiding axonal migration and synaptogenesis (Fride et al., 2009). In contrast, the function of CB2 in the developing brain is poorly understood. It is known that mHFD may elevate CB2 in the offspring’s brain (Dias-Rocha et al., 2018). As CB2 is known to attenuate inflammation (Turcotte et al., 2016), it is suggested that it can be linked to neurodevelopment through the modulation of microglial function (Lisboa et al., 2016); however, the mechanism of CB2 dysregulation by mHFD has not been described yet.

It has been shown in rodents that mHFD epigenetically dysregulates the expression of CB1 gene *Cnr1* by histone acetylation, DNA methylation, and/or specific miRNAs (Almeida et al., 2019; Gawliński et al., 2021). In male offspring, mHFD decreases *Cnr1* expression in the PFC and in the dorsal striatum (DS), but increases in the hippocampus and hypothalamus (Almeida et al., 2019; Gawliński et al., 2021). As a consequence, the expression of CB1 protein is also increased, as has been demonstrated in the hypothalamus of mHFD offspring (Dias-Rocha et al., 2018). It is suggested that mHFD dysregulates the expression of *Cnr1* by differential DNA methylation of CpG islands at the *Cnr1* promoter. In particular, the *Cnr1* promoter is hypermethylated in the PFC and hypomethylated in the hippocampus of mHFD offspring, in line with observed differences in *Cnr1* mRNA expression (Gawliński et al., 2021). However, no changes in DNA methylation have been observed in the DS, indicative of other epigenetic mechanisms that can contribute to the downregulation of *Cnr1* expression by mHFD. For example, in the hypothalamus of mHFD offspring, *Cnr1* expression is upregulated by elevated histone acetylation on the *Cnr1* promoter (Almeida et al., 2019). Whether histone acetylation contributes to *Cnr1* expression in other brain regions remains to be defined. In addition to the transcriptional control by DNA methylation and histone modifications, the expression of CB1 can be further adjusted post-transcriptionally due to *Cnr1* mRNA interaction with inhibiting miRNAs. It has been shown that *miR-212-5p* and *miR-154-3p*, which putatively target *Cnr1* transcript (Chen and Wang, 2020), are elevated in the DS, but downregulated in the hippocampus of mHFD offspring, in line with observed aberrations of *Cnr1* expression (Gawliński et al., 2021). In contrast, in the PFC, only *miR-212-5p*, but not *miR-154-3p*, is overexpressed, further indicating the heterogeneity of epigenetic regulation of *Cnr1* expression in the brain. Interestingly, *Cnr1* expression differences were more pronounced in postnatal and juvenile pups of HFD dams and mostly normalized by adulthood (except for the hippocampus) (Dias-Rocha et al., 2018; Almeida et al., 2019; Gawliński et al., 2021), possibly due to waning epigenetic inhibition with age in the absence of a positive feedback mechanism, as described for BDNF (see above). Furthermore, CB1 expression appears to be unaffected in mHFD female offspring, as normal levels of both CB1 protein and *Cnr1* mRNA were found throughout the brain (Dias-Rocha et al., 2018; Almeida et al., 2019; Gawliński et al., 2021). This further supports the notion that male offspring seem to be more sensitive to mHFD, as demonstrated by different studies, ranging from molecular to behavioral investigations.

## Alterations in offspring behavior caused by maternal high-fat diet

Previously described mHFD-induced changes in brain morphology, maternal and offspring microbiome as well as epigenetic regulation are reflected in offspring behavioral phenotype. Human studies have shown that the children of overweight and obese mothers have an increased risk of developing ASD (Krakowiak et al., 2012; Moss and Chugani, 2014; Reynolds et al., 2014b). In animal models, mHFD also causes changes in offspring behavior, associated with ASD symptoms in humans, such as social behavior deficits (Kang et al., 2014; Buffington et al., 2016; Liu et al., 2021), and impaired cognitive functions (Bilbo and Tsang, 2010; Tozuka et al., 2010; Page et al., 2014; Graf et al., 2016; Fusco et al., 2019; Liu et al., 2021). Furthermore, human studies associate maternal obesity with ADHD in their children (Rodriguez et al., 2008; Rodriguez, 2010; Chen et al., 2014b; Field, 2014). Animals’ mHFD models mimic the hyperactivity observed in ADHD cases by increased locomotion activity (Tozuka et al., 2010; Kang et al., 2014; Page et al., 2014; Balsevich et al., 2016; Lippert et al., 2020) and higher repetitive compulsive-like behavior (Buffington et al., 2016; Bordeleau et al., 2022). Moreover, human studies suggest that maternal consumption of HFD during pregnancy and lactation increases the risk of anxiety and depression in children and adolescents (Rodriguez, 2010; Van Lieshout et al., 2013). Animal research provides further evidence that mHFD increases anxiety-like behavior in offspring (Peleg-Raibstein et al., 2012; Kang et al., 2014; Buffington et al., 2016; Glendining et al., 2018). mHFD may also lead to the development of schizophrenia (Schaefer et al., 2000; Kawai et al., 2004; Khandaker et al., 2012; Rivera et al., 2015), which is related to impaired processing and integration of sensory and motor information (Braff et al., 2001; Rascle et al., 2001), as has been observed in animal studies using mHFD models (Wolfrum and Peleg-Raibstein, 2019; Zieba et al., 2019; Bordeleau et al., 2021). In this section, we reviewed mouse behavioral phenotypes induced by mHFD, such as offspring deficits in cognition, sociability, anxiety, locomotive activity, and sensorimotor gating (Table 1). We have defined animals younger than 3 weeks as postnatal, juveniles as 3–8 weeks of age, young adults as 9–13 weeks of age, mature adults as 14–26 weeks, and aged animals as older than 27 weeks.

**TABLE 1:** Maternal high-fat diet (mHFD) outcome for offspring behavior in mice.

Offspring					Maternal diet			References
Effect	Age	Sex	Test	Behavioral phenotype of mHFP offspring	Duration (weeks before pregnancy + period)	Start (weeks)	Fat in diet, CD HFD	
Cognition	5.5, 13, and 80 weeks	Both	Y-maze	No effect on working memory in both sexes	3 + gestation + lactation	Not specified	Normal chow 60%	Wolfrum and Peleg-Raibstein, 2019
	38 weeks	Male	Y-maze	No effect on working memory	5 + gestation + lactation	5	10.5% 58%	Zieba et al., 2019
	5.5, 13, and 80 weeks	Both	T-maze	↓ spatial learning in both sexes at 13 weeks	3 + gestation + lactation	Not specified	Normal chow 60%	Wolfrum and Peleg-Raibstein, 2019
	4 and 11 weeks	Male	Barnes maze	↓ spatial learning at 4 weeks	6 + gestation + lactation (until day 16)	5	10.6% 57.5%	Tozuka et al., 2010
	4–12 weeks	Male	MWM	↓ spatial learning	4 + gestation + lactation (until day 14)	4.2	6.55% 60%	Fusco et al., 2019
			NORT	↓ recognition memory				
	8–10 weeks	Both	Y-maze	↓ working memory in both sexes	12 + gestation + lactation	7	16.7% 60%	Liu et al., 2021
			NORT	↓ recognition memory in both sexes				
			3CT	↓ social memory in both sexes				
	16 weeks	Both	Y-maze	No effect in both sexes	8 + gestation + lactation	4	10% 60%	Graf et al., 2016
			NORT	↓ recognition memory in males				
	4.5 weeks	Both	3CT	↓ social memory in males	4 + gestation + lactation	6–7	Normal chow 60%	Bordeleau et al., 2021
	7–12 weeks	Male	3CT	↓ social memory	8 + gestation + lactation	Not specified	13.4% 60%	Buffington et al., 2016
Sociability	P8	Both	USV	↓ latency to call in males; ↑ number of ultrasonic vocalizations in males; ↑ loudness of the calls in females	8 + gestation + lactation	Not specified	10% 45%	Glendining et al., 2018
	4.5 weeks	Both	3CT	No effect on sociability in both sexes	4 + gestation + lactation	6–7	Normal chow 60%	Bordeleau et al., 2021
	5.5–6 weeks	Both	3CT	↓ sociability in females	6 + gestation + lactation	6	10% 60%	Kang et al., 2014
	8–10 weeks	Both	3CT	↓ sociability in both sexes	12 + gestation + lactation	7	16.7% 60%	Liu et al., 2021
	7–12 weeks	Male	3CT	↓ sociability	8 + gestation + lactation	Not specified	13.4% 60%	Buffington et al., 2016
			RSI	↓ sociability				
	38 weeks	Male	RSI	No effect on sociability	5 + gestation + lactation	4	13% 43%	Zieba et al., 2019
Anxiety	4.5–5 weeks	Both	OFT	↑ anxiety-like behavior in females	6 + gestation + lactation	6	10% 60%	Kang et al., 2014
	5 weeks	Both	EPM	No effect on anxiety in both sexes	4 + gestation + lactation	6–7	Normal chow 60%	Bordeleau et al., 2021
	7–12 weeks	Male	OFT	↑ anxiety-like behavior	8 + gestation + lactation	Not specified	13.4% 60%	Buffington et al., 2016
			Marble burying	↑ compulsive-like behavior				
	8.5–12 weeks	Both	Marble burying	↑ compulsive-like behavior in both sexes	4 + gestation + lactation	6	Normal chow 60%	Bordeleau et al., 2022
	Adult	Both	OFT	No effect on anxiety in both sexes	3 + gestation + lactation	12–13	Normal chow 60%	Peleg-Raibstein et al., 2012
			EPM	↑ anxiety-like behavior in both sexes				
	17 weeks	Both	EPM	↑ anxiety-like behavior in females	8 + gestation + lactation	Not specified	10% 45%	Glendining et al., 2018
	13 and 52 weeks	Male	OFT	↓ anxiety-like behavior at 13 weeks; ↑ anxiety-like behavior at 52 weeks	6 + gestation (E18 ± 2)	5	10.5% 58%	Balsevich et al., 2016
			EPM	↓ anxiety-like behavior at 13 weeks; ↑ anxiety-like behavior at 52 weeks				
	26 weeks	Both	EZM	No effect on anxiety in both sexes	Lactation	14	Normal chow 60%	Lippert et al., 2020
	38 weeks	Male	OFT	No effect on anxiety	5 + gestation + lactation	4	13% 43%	Zieba et al., 2019
			EPM	No effect on anxiety				
			NSF	No effect on feeding behavior in novel environment				
Locomotive activity	4.5–5 weeks	Both	OFT	↑ activity in males	6 + gestation + lactation	6	10% 60%	Kang et al., 2014
	3 and 10 weeks	Male	OFT	↑ activity at 10 weeks	6 + gestation + lactation (until day 16)	5	10.6% 57.5%	Tozuka et al., 2010
	13 and 52 weeks	Male	OFT	↑ activity at 13 weeks	6 + gestation (E18 ± 2)	5	10.5% 58%	Balsevich et al., 2016
	26 weeks	Both	OFT	↑ activity in males	Lactation	14	Normal chow 60%	Lippert et al., 2020
	38 weeks	Male	OFT	No effect on locomotion	5 + gestation + lactation	5	10.5% 58%	Zieba et al., 2019
	5.5, 13, and 80 weeks	Both	OFT	No effect on locomotion in both sexes	3 + gestation + lactation	Not specified	Normal chow 60%	Wolfrum and Peleg-Raibstein, 2019
Sensorimotor gating	5.5 weeks	Both	PPI	Normal startle reflex in both sexes; ↓ prepulse inhibition in both sexes	4 + gestation + lactation	6–7	Normal chow 60%	Bordeleau et al., 2021
	38 weeks	Male	PPI	↑ startle reflex; ↑ prepulse inhibition	5 + gestation + lactation	5	10.5% 58%	Zieba et al., 2019
	5.5, 13, and 80 weeks	Both	PPI	↑ prepulse inhibition in box sexes at 13 weeks; ↑ prepulse inhibition in box sexes at 80 weeks	3 + gestation + lactation	Not specified	Normal chow 60%	Wolfrum and Peleg-Raibstein, 2019

CD, control diet; HFD, high-fat diet; mHFD, maternal high-fat diet; NORT, novel object recognition test; MWM, Morris water maze; 3CT, three-chamber test; RSI, reciprocal social interaction; OFT, open field test; EPM, elevated plus-maze; USV, ultrasonic vocalization; EZM, elevated zero maze; NSF, novelty suppressed feeding; PPI, prepulse inhibition; FC, fear conditioning; E, embryonic day; P, postnatal day.

### The impact of maternal high-fat diet on offspring cognition

In mice, cognition can be evaluated by tests accounting for different aspects of learning and memory, such as working or long-term memory, spatial, recognition, or social memory, as well as cognitive flexibility. For the technical aspects of the Barnes maze, Morris water maze (MWM), Y-maze, T-maze, three-chamber test (3CT) as well as novel object recognition test (NORT), please refer to previous publications (Deacon and Rawlins, 2006; Vorhees and Williams, 2006; Yang et al., 2011; Leger et al., 2013; Cordner and Tamashiro, 2015; Kraeuter et al., 2019).

Several groups have shown that mHFD does not affect the performance of juveniles, young adults, or aged offspring of both sexes in the Y-maze, indicating normal working memory in a spatial context (Graf et al., 2016; Wolfrum and Peleg-Raibstein, 2019; Zieba et al., 2019). In contrast, Liu et al. (2021), who used an mHFD model with a particularly long period of HFD before pregnancy, found deficits in Y-maze performance in both male and female young adult mice, suggesting that the duration of HFD may be critical to the outcome to the offspring (Liu et al., 2021).

In contrast to the working memory, long-term memory appears to be consistently impaired in both male and female mHFD juvenile and adult offspring (Graf et al., 2016; Fusco et al., 2019; Liu et al., 2021). mHFD offspring demonstrates a reduced preference for novel objects in NORT and a reduced preference for a stranger mouse in 3CT (Buffington et al., 2016; Graf et al., 2016; Fusco et al., 2019; Bordeleau et al., 2021; Liu et al., 2021).

In line with long-term memory deficits, mHFD has also been shown to impair spatial learning (Tozuka et al., 2010; Fusco et al., 2019). Juvenile males born to HFD dams demonstrate higher latency of reaching hidden escape box or underwater platform during training in Barnes maze and MWM, respectively (Tozuka et al., 2010; Fusco et al., 2019). Interestingly, no effect on spatial learning in the Barnes maze has been observed at 11 weeks, indicating that cognition deficits may coincide with particular neurodevelopmental stages and may be only temporary (Tozuka et al., 2010). Such a hypothesis is further supported by T-maze performance, which was impaired only in young adults, but not in juvenile or aged mHFD offspring of both sexes (Wolfrum and Peleg-Raibstein, 2019).

Observed learning and memory deficits in mHFD offspring were suggested to stem from a decreased level of BDNF in the hippocampus (Tozuka et al., 2010; Fusco et al., 2019) or decreased myelination in the medial PFC (Graf et al., 2016). However, other mechanisms are likely to be involved, particularly, due to the quite specific nature of observed deficits and their variability with age and sex (Tozuka et al., 2010; Graf et al., 2016; Wolfrum and Peleg-Raibstein, 2019; Bordeleau et al., 2021). At the moment, mHFD effects have not been evaluated for cognitive flexibility, which would further help us to understand how learning and memory deficits arise in the offspring of obese mothers.

### Maternal high-fat diet in the programming of social behaviors

Mice are social animals that participate in complex interactions with each other, and it is known that neurodevelopmental deficits in mice are reflected in their aberrant social behavior (Shemesh et al., 2013; Park et al., 2022). The sociability in mice is usually determined either through the evaluation of reciprocal social interaction (RSI) or in a three-chamber test (3CT) for the social approach. Reciprocal social interactions are evaluated by quantifying social behaviors, such as nosing, anogenital sniffing, crawling, following, and allogrooming (Silverman et al., 2010). In a 3CT, the sociability is determined as a preference given to another mouse, which is known to have normal social behavior, compared to an inanimate object (Yang et al., 2011).

Young adult mHFD offspring, both males and females, have demonstrated reduced sociability in both 3CT and RSI (Buffington et al., 2016; Liu et al., 2021). In contrast, there were no social deficits identified in mHFD offspring post-weaning (4.5 weeks) (Bordeleau et al., 2021), while only females demonstrated reduced sociability at juvenile age (Kang et al., 2014). It is known that female neurodevelopment precedes that of males (Holder and Blaustein, 2014), therefore, such sexual dimorphism of social behavior could be explained by the differences in the timing of maturation of critical brain areas. Interestingly, both male and female mHFD-aged offspring demonstrated normal reciprocal social interactions (Zieba et al., 2019), suggesting that social deficits induced by mHFD may be transient during certain developmental periods and be normalized later in life.

Importantly, social deficits in mHFD offspring appear to be mediated through the gut microbiota-brain axis and can be restored by oral treatment by supplementing the offspring with deficient microorganisms, such as *L. reuteri* (Buffington et al., 2016). As described above, gut microbiota dysbiosis may interfere with normal neurodevelopment due to increased inflammation. Interestingly, the level of pro-inflammatory cytokines in the offspring’s brain can be reduced by switching the dams from HFD to CD during lactation (Kang et al., 2014). The reduced inflammatory milieu is followed by restored sociability, suggesting that lactation may be a critical period for sociability development (Kang et al., 2014).

As social deficits induced by mHFD appear to develop postnatally, it is also important to evaluate early postnatal offspring neurodevelopment. Unfortunately, such tests as 3CT and RSI are only suitable to measure sociability post-weaning and cannot be used to evaluate early postnatal behavior. To evaluate neurodevelopmental profiles that have been developed prenatally and during lactation, we need other tools of behavioral phenotyping, such as the quantification of ultrasonic vocalizations (USV) (Scattoni et al., 2009). Using USV measurements, it has been shown that mHFD promotes enhanced vocal communication in offspring (Glendining et al., 2018). Postnatal mHFD males exhibit decreased latency to call and emit more vocalizations, while females vocalize louder (Glendining et al., 2018). USV aberrations have been observed in different models of neurodevelopmental disorders (Scattoni et al., 2009); however, exact mechanisms of how vocalizations are affected by mHFD are yet to be described.

### Maternal high-fat diet in the programming of anxiety

Neurodevelopmental disorders are often associated with increased anxiety, which may also be induced by mHFD. Two main tests are used to measure anxiety in rodents. The open-field test (OFT) evaluates how much time a mouse spends and/or travels in the inner zone of the open arena compared to the outer zone (Seibenhener and Wooten, 2015). In the EPM test, the mouse can choose to spend time either in open or closed arms (Bouwknecht and Paylor, 2008). In both of these tests, the natural exploratory instinct of the mouse competes with the anxiousness to enter open areas. It is believed that reduced time spent in the center of the OFT arena or in the open arms of EPM is indicative of increased anxiety (Bouwknecht and Paylor, 2008; Seibenhener and Wooten, 2015).

Importantly, the mHFD effect on anxiety-like behavior appears to be particularly variable (Table 1). Some researchers have reported increased anxiety in juvenile and adult female, but not male mHFD offspring (Kang et al., 2014; Glendining et al., 2018), while others failed to observe such sexual dimorphism (Peleg-Raibstein et al., 2012) or have not identified any differences in anxiety levels altogether (Peleg-Raibstein et al., 2012; Lippert et al., 2020; Bordeleau et al., 2021). Anxiety-like behaviors are more noticeable in juveniles (Kang et al., 2014; Buffington et al., 2016) and aged (Balsevich et al., 2016) mHFD mice and appear to normalize in adults mHFD offspring (Zieba et al., 2019; Lippert et al., 2020). This may suggest that anxiety-like behavior becomes more pronounced during periods of higher neuronal plasticity and could correspond to microglial activation. However, the interpretation of the results of anxiety testing is further complicated by differences observed between OFT and EPM tests (Peleg-Raibstein et al., 2012). The development of anxiety-like behavior, similarly to social deficits described above, appears to be tightly linked to maternal diet during lactation. For example, switching the dams from HFD to CD at the end of gestation induces an opposite phenotype of reduced anxiety levels in young adults (Balsevich et al., 2016). Likewise, anxiety-like behavior was normalized in juvenile mHFD females, when the maternal diet was switched to CD during lactation (Kang et al., 2014). In contrast, mHFD restricted only to the period of lactation may not be sufficient to induce any anxiety-like deficits in the offspring (Lippert et al., 2020).

Some studies have used additional tests to evaluate the anxiety levels in adult mHFD offspring, such as elevated zero mazes (EZM) (Lippert et al., 2020) and novelty-suppressed feeding (Zieba et al., 2019), but they have not identified any mHFD effects in adult offspring. In addition, anxiety-like behavior is sometimes associated with the compulsive burying of unharmful objects, such as in the marble-burying test (Taylor et al., 2017). mHFD has been shown to increase the number of buried marbles by young adults, indicative of repetitive compulsive-like behavior (Buffington et al., 2016; Bordeleau et al., 2022) and further supports the notion that mHFD can lead to anxiety-like behaviors in young offspring. As anxiety responses are mostly mediated by the amygdala, unsurprisingly mHFD has been shown to elevate inflammation and disturb GABAergic and serotonergic systems in the amygdala of the offspring (Peleg-Raibstein et al., 2012; Kang et al., 2014; Glendining et al., 2018).

### The impact of maternal high-fat diet on locomotion activity

To determine rodent behavioral phenotypes, related to hyperactivity, locomotor activity is often evaluated by measuring the speed and the traveled distance of the animals allowed to freely explore an open field arena (Seibenhener and Wooten, 2015). In line with human studies that link mHFD to hyperactivity, it has been shown that mHFD also leads to hyperactivity in mouse models (Tozuka et al., 2010; Kang et al., 2014; Balsevich et al., 2016; Lippert et al., 2020). Interestingly, hyperactivity phenotype has been only indicated in male offspring (Kang et al., 2014; Lippert et al., 2020), mirroring a higher prevalence of hyperactivity diagnosed in boys compared to girls (Ramtekkar et al., 2010). Increased locomotion was observed in mHFD juveniles and young adults, but not aged individuals (Balsevich et al., 2016; Zieba et al., 2019), suggesting that hyperactivity phenotype can be restored with age.

It appears that the duration of mHFD before pregnancy can be critical for the mHFD effect on the locomotive phenotype of the offspring, as Wolfrum and Peleg-Raibstein (2019) did not identify any elevation locomotor activity in a model, in which the dams were fed HFD for as little as 3 weeks before pregnancy. Such a short duration of mHFD may not be sufficient to cause maternal metabolic disturbances, which in turn affect offspring neurodevelopment *via* previously described mechanisms.

Hyperactivity can be induced by the disbalanced serotonin and dopamine signaling and mHFD has been shown to alter serotonergic and dopaminergic circuitry (Sullivan et al., 2015; Lippert et al., 2020); however, further studies are required to define whether elevated inflammatory milieu directly contributes to the development of hyperactive phenotype.

### The impact of maternal high-fat diet on sensorimotor performance

In addition to locomotion, mHFD has also been shown to impair the sensorimotor performance in the offspring. Alterations in sensorimotor performance are commonly observed in neurodevelopmental disorders and can be measured by such tests as prepulse inhibition (PPI) of the acoustic startle response test (Buuse et al., 2003; San-Martin et al., 2018).

In animal models, transient reduction of PPI has been observed in juvenile mHFD males and females (Bordeleau et al., 2021), which was reversed in adult and aged animals (Wolfrum and Peleg-Raibstein, 2019; Zieba et al., 2019). In contrast, startle reflex has been shown to be normal in mHFD juveniles, but has been found to be increased in aged individuals (Zieba et al., 2019; Bordeleau et al., 2021). As with the locomotive phenotype, impaired sensorimotor performance may be associated with aberrant dopamine signaling in the striatum (Peleg-Raibstein et al., 2012). Furthermore, elevated startle response can also be indicative of increased anxiety (Pantoni et al., 2020).

## Discussion

The increasing prevalence of obesity among women of reproductive age highlights the importance of understanding how diet-induced changes in maternal metabolism, microbiota, and inflammatory status shape the neurodevelopment of the offspring. Both retrospective human studies and those including animal models indicate that maternal obesity has long-term negative effects on the function of the offspring’s brain. In addition to the behavioral and molecular phenotypes identified in the mHFD offspring, multiple publications have reported that maternal obesity leads to aberrant feeding patterns and disturbed metabolic programming in the offspring. Such outcomes of mHFD were beyond the scope of this review as there are other comprehensive publications that have focused on this particular aspect of maternal nutrition (Chaves et al., 2021; Gawlińska et al., 2021; Larsen and Bode, 2021).

For a long time, the studies of mHFD effects, as any other animal studies, have included only male offspring, thus limiting our understanding of the consequences of maternal obesity on the neurodevelopment of females. There is accumulating evidence that neurodevelopment is sexually dimorphic and may include different molecular pathways and mechanisms. This includes distinct behavioral phenotypes as well as the variability among neuroinflammatory responses, epigenetic regulation, or gene expression patterns both in human disease and in animal models. Recent scientific policy changes by the funding bodies promote and/or require including both male and female animals in biological studies, therefore most recent publications started describing female phenotype as well; however, the effect of mHFD on the female brain remains under-investigated. Nevertheless, current data suggest that the neurodevelopment of males may be more sensitive to maternal diet and present larger phenotypic effects compared to females. The apparent robustness of female neurodevelopment may be associated with the protection of female hormones. Yet differences in hormonal milieu cannot explain prenatal and early postnatal differences that are observed between males and females in mHFD models, thus this question remains to be investigated.

Neurodevelopmental outcomes of mHFD are often assessed through behavioral phenotyping of animals. Even though rodent models of mHFD seem to mimic human disease phenotypes quite properly, the results of animal behavior tests should be evaluated with caution, as it often depends on both the sex and age of the animal as well as the timeline of mHFD model implementation. In particular, heterogeneity of behavioral consequences appears to result from the different duration of mHFD before pregnancy as well as its continuance after the birth of the offspring. In humans, mHFD may often precede the pregnancy for a significant period, with dietary habits acquired even prepubertally, thus animal models that include shorter periods of mHFD may only be suitable to evaluate acute effects of elevated fat content in the maternal diet rather than long-term effects related to obesity and metabolic disorder.

While in the human population mHFD and resulting obesity are difficult to separate, using animal models that are less prone to HFD-induced obesity, may help to isolate the direct effects of mHFD in contrast to those resulting from maternal obesity. For example, such mouse lines as SWR/J, A/J, or BALB/c are more resistant to the effects of dietary fat, including weight gain, hyperinsulinemia, hyperglycemia, and hyperleptinemia (Eberhart et al., 1994; Parekh et al., 1998; Bahceci et al., 1999; Leibowitz et al., 2004, 2005; Boi et al., 2016). Furthermore, genetically diverse collaborative cross mouse strains, such as 129S1/SvImJ, PWK/EiJ, CAST/PhJ, or WSB/EiJ, demonstrate variable phenotypic responses to HFD, comparison of which could also be used to distinguish mHFD-specific outcomes. Such an approach has been used to compare fetal development in diet-sensitive and diet-resistant HFD dams in a non-human primate model (Grant et al., 2011). Finally, targeted genetic modifications have been used to develop obesity-resistant mouse models, that allow for the investigation of more specific aspects of mHFD (Burrage et al., 2010; Lo et al., 2010; Zhao et al., 2020b).

Interestingly, lactation appears to be a critical period for the development of certain behavioral deficits that can be rescued if dams are reversed to the normal diet after giving birth. These findings suggest possible intervention strategies that could attenuate the effects of maternal diet on their children. Other possible interventions could include the supplementation of the offspring with lacking microorganisms to restore the necessary microbiota composition that supports normal neurodevelopment. However, it is important to note that studies, which use probiotic treatment to counteract mHFD-induced microbiota disbalance, rarely evaluate whether the intervention ameliorated the dysbiosis. Furthermore, probiotic supplementation has been demonstrated to have behavioral effects even in the absence of dysfunctional gut microbiota (Tillisch et al., 2013; Wang et al., 2016; Bagga et al., 2018). Attenuation of chronic inflammation may also restore a more beneficial environment *in utero*, helping to rescue neurodevelopmental deficits. However, most of the intervention research is performed on animal models and we are in pressing need of human studies to help us to identify possible strategies to counteract neurodevelopmental fallout for our young generations due to the obesity pandemic advancing worldwide.

## Author contributions

GU, AK, FB, and AS: writing – original draft. GU and UN: writing – review and editing. UN: supervision. All authors contributed to the article and approved the submitted version.

## Conflict of interest

The authors declare that the research was conducted in the absence of any commercial or financial relationships that could be construed as a potential conflict of interest.

## Publisher’s note

All claims expressed in this article are solely those of the authors and do not necessarily represent those of their affiliated organizations, or those of the publisher, the editors and the reviewers. Any product that may be evaluated in this article, or claim that may be made by its manufacturer, is not guaranteed or endorsed by the publisher.
